# How do gender and disability influence the ability of the poor to benefit from pro-poor health financing policies in Kenya? An intersectional analysis

**DOI:** 10.1186/s12939-018-0853-6

**Published:** 2018-09-19

**Authors:** Evelyn Kabia, Rahab Mbau, Kelly W. Muraya, Rosemary Morgan, Sassy Molyneux, Edwine Barasa

**Affiliations:** 1 Health Economics Research Unit, KEMRI-Wellcome Trust Research Programme, P.O. Box 43640-00100, Nairobi, Kenya; 2 Health Systems & Research Ethics Department, KEMRI-Wellcome Trust Research Programme, P.O. Box 43640-00100, Nairobi, Kenya; 3 John Hopkins Bloomberg School of Public Health, 615 North Wolfe Street, Baltimore, MD 21205 USA; 4 Health Systems & Research Ethics Department, KEMRI-Wellcome Trust Research Programme, P.O. Box 230-80108, Kilifi, Kenya; 5 Nuffield Department of Medicine, University of Oxford, Oxford, UK

**Keywords:** Gender, Disability, Poverty, Intersectionality, Pro-poor, Kenya

## Abstract

**Background:**

Health inequity has mainly been linked to differences in economic status, with the poor facing greater challenges accessing healthcare than the less poor. To extend financial coverage to the poor and vulnerable, Kenya has therefore implemented several pro-poor health policy reforms. However, other social determinants of health such as gender and disability also influence health status and access to care. This study employed an intersectional approach to explore how gender disability and poverty interact to influence how poor women in Kenya benefit from pro-poor financing policies that target them.

**Methods:**

We applied a qualitative cross-sectional study approach in two purposively selected counties in Kenya. We collected data using in-depth interviews with women with disabilities living in poverty who were beneficiaries of the health insurance subsidy programme and those in the lowest wealth quintiles residing in the health and demographic surveillance system. We analyzed data using a thematic approach drawing from the study’s conceptual framework.

**Results:**

Women with disabilities living in poverty often opted to forgo seeking free healthcare services because of their roles as the primary household providers and caregivers. Due to limited mobility, they needed someone to accompany them to health facilities, leading to greater transport costs. The absence of someone to accompany them and unaffordability of the high transport costs, for example, made some women forgo seeking antenatal and skilled delivery services despite the existence of a free maternity programme. The layout and equipment at health facilities offering care under pro-poor health financing policies were disability-unfriendly. The latter in addition to negative healthcare worker attitudes towards women with disabilities discouraged them from seeking care. Negative stereotypes against women with disabilities in the society led to their exclusion from public participation forums thereby limiting their awareness about health services.

**Conclusions:**

Intersections of gender, poverty, and disability influenced the experiences of women with disabilities living in poverty with pro-poor health financing policies in Kenya. Addressing the healthcare access barriers they face could entail ensuring availability of disability-friendly health facilities and public transport systems, building cultural competence in health service delivery, and empowering them to engage in public participation.

## Background

Universal health coverage (UHC) is recognized as an avenue for improving equity in health [1] and Kenya has made a commitment to achieve UHC by the year 2022 [2]. Achieving UHC will strengthen Kenya’s vision 2030 social pillar by improving access to affordable and quality healthcare for all Kenyans [3, 4]. To improve access to care, the Kenyan government has made key policy choices. These include using tax funding to subsidize service provision in public health facilities and scaling up contributory health insurance through the National Hospital Insurance Fund (NHIF) to cover all Kenyans [3]. However, a key challenge is the country’s poverty rate of 36.1% as at 2015/2016 [5].

To extend financing coverage to the poor and vulnerable, Kenya has implemented several pro-poor health policy reforms since 2013. These include: 1) introduction of a free maternity policy [6]; 2) abolition of user fees in public primary healthcare facilities (dispensaries and health centres) and; 3) introduction of a health insurance subsidy programme (HISP) for the poor where the government fully subsidises the cost of NHIF premiums for the poorest households in Kenya enabling them to access both inpatient and outpatient care at public, low-cost private and faith-based facilities [7]. Pro-poor health financing systems ensure that contributions to healthcare costs are based on people’s ability to pay; they offer financial risk protection to the poor and enhance access to quality healthcare services [5]. However, if not well designed, policy reforms aimed at achieving UHC, even those specifically targeted at the poor, may preferentially benefit the well-off while excluding the poor resulting in inequitable health systems [1, 8, 9].

The World Health Organization in 2008 declared health equity as a new global agenda placing emphasis on how social determinants of health (SDH), shaped by the allocation of resources and power at micro, meso, and macro levels, interact to produce health inequities, most of which can be avoided [10]. SDH such as gender, age, education, race/ethnicity, geographical location, class, and occupation influence health outcomes [11]. Intersectionality also fosters an understanding that people’s lives are complex, they consist of multiple dimensions and lived experiences, and are shaped by the interaction of various SDH [12, 13]. These interactions take place within interconnected power structures and systems (e.g. politics, governments, policies, religion) which lead to people experiencing various forms of privilege and oppression [12]. Intersectionality has been appreciated as an essential framework for understanding and addressing inequities in health [13].

Health inequity has mainly been linked to differences in economic status, with poorer people facing greater challenges accessing healthcare and reporting poorer health outcomes than the less poor [14–16]. However, with regards to intersectional theory, inequities do not result from individual and independent factors but they are the result of intersections of various social determinants of health, experiences and power structures [12]. For example, women are more likely to experience poor health than men [17], which suggests that inequities in health cannot be fully explained by people’s socio-economic status [14]. Disability is another SDH that influences access to equitable care [18]. According to the International Classification of Functioning, Disability and Health, disability is described as “an umbrella term for impairments, activity limitations or participation restrictions,” proposing that “a person’s functioning and disability is a dynamic interaction between health conditions (diseases, disorders, injuries, traumas, etc.) and contextual factors,” [19]. Globally, over 1000 million people live with a disability with close to 80% living in developing countries [20]. As at 2015/2016, 2.8% (45,371) of the Kenyan population was living with a disability. 1% had vision disability, 0.5% hearing disability, 0.2% speech disability, 1.0% physical disability, 0.4% mental disability, 0.1% self-care disability and 0.1% had other types of disability [26]. The Kenya Persons with Disabilities Act of 2003 stipulates that the National Council for Persons with Disabilities (NCPWD) should be represented during the implementation of the Ministry of Health programs [27]. The Act also advocates for the availability of; affordable health services; healthcare personnel and disability-friendly environments that facilitate persons with disabilities access to assistive devices, buildings, and transport systems that enhance their mobility [27]. Globally, women, older persons and the poor are disproportionately affected by disability [20]. In addition, a relationship exists between disability and poverty [20] with evidence showing that globally, the rates of poverty, unemployment, low education levels are higher among people with disabilities [18]. People with disabilities also face various healthcare access barriers and despite having increased need for health services [17, 21–23], their needs are more likely not to be met [23–25] leading to poorer health outcomes compared to people without disability [20].

Despite increased awareness of SDH and their contribution to health inequities [11] there is a dearth of literature addressing these complex interactions. This study is part of a larger study that examined perceptions and experiences of the poor in Kenya with health financing mechanisms that target them. In this paper, an intersectional approach was employed to explore how gender (being a woman ), disability and poverty intersect to influence how women with disabilities living with poverty in Kenya benefit from pro-poor health financing policies. Incorporating an intersectional lens will inform the design of equitable health policies by enhancing policy makers understanding of the varying degrees of vulnerability across social groups.

## Methods

### Study setting

Kenya has a devolved government system consisting of a national government and 47 county governments which function through interdependent relationships. Health service delivery falls under the mandate of the county government [28]. The healthcare system in Kenya is organized into 4 tiers: Tier 1- community, Tier 2-primary care which comprises dispensaries, health centers and clinics, Tier 3-secondary referral which comprises county hospitals, and Tier 4-tertiary referral which comprises national referral hospitals [28].

This study was conducted in two purposely selected counties in Kenya. The counties were selected based on two criteria; 1) the presence of a health and demographic surveillance system (HDSS), and 2) being either a rural or urban county. Counties with HDSS sites were selected because they regularly collect information on household socio-economic status and they rank households according to their wealth. This was important because our study purposed to collect data from the poor and the HDSS offered an opportunity to identify individuals living in poverty to be included in the study. Data were also collected from beneficiaries of the Health Insurance Subsidy Programme (HISP) for the poor. The HISP programme selects beneficiaries from the Kenyan Government poverty list that is developed and maintained by the Ministry of Labour, Social Security, and Services. Poverty identification for inclusion in the poverty list is carried out by proxy-means testing and verification by the local community [29]. To avoid the potential for identification of the study counties they were labeled County A (urban) and County B (rural). Table 1 outlines the demographic and health indicators of the selected counties. County A was highly populated compared to County B but there was an almost equal gender distribution across the two counties. The prevalence of disability, morbidity, home deliveries and poverty was higher in County B compared to County A. In addition, the prevalence of disability and morbidity in County B was above the national average. With regards to people residing in DHSS sites, the rural HDSS was highly populated compared to the urban HDSS since it covered a wider area. In terms of health facility coverage, County A had a relatively higher number of health facilities compared to county B reflecting a pro-urban distribution of health facilities. With regards to health financing, the total county government spending on health was  higher in County A compared to County B and health insurance coverage in County A was  also five times greater than that in county B.

**Table 1:** County demographic and health indicators

Indicator	County A (Urban)	County B (Rural)	Country
Population 2015/2016 [26, 30]			
Total	4,463,000	985,000	45,371,000
Male	2,237,000 (50.1%)	466,000 (47.3%)	22,393,000 (49.4%)
Female	2,226,000 (49.9 %)	519,000 (52.7%)	22,977,000 (50.6%)
Population with any disability	1.2%	5.3%	2.8%
Morbidity	19.2%	33.2%	21.5%
Poverty rate	16.7%	33.8%	36.1%
Home deliveries for under 5	8.8%	13%	31.3%
HDSS			
HDSS residents	63,639 [31]	255,000 [32]	824,595 [31–36]
Health facilities in 2015 [37, 38]			
Public	161	123	4,929
Nongovernmental	118	7	347
Faith-based	100	16	1,081
Private	543	28	3,797
Health Financing			
Total government health spending (per capita, KES) (2015) [37, 38]	1,745	1,495	1,585
Health insurance coverage (2015/2016) [26]	40.7%	7.6%	19.0%

### Study design and data collection

This study employed a qualitative cross-sectional approach. A total of eight focus group discussions (FGDs) and thirty in-depth interviews (IDIs) were conducted. In each county, people living in poverty were selected purposively from a list of households in the lowest wealth quintile in the HDSS and a list of HISP beneficiaries. Study participants were identified with the assistance of HDSS coordinators, NHIF and Social Services officials in each study county. Maximum variation sampling was used to ensure representation across gender and various age groups. However, this paper presents findings from 11 in-depth interviews (5 in County A and 6 in County B) conducted with women with disabilities living in poverty in the two study counties. This sub-group of the larger study population was identified purposively based on two criteria, 1) being a woman and, 2) having a disability, in addition to being in the lowest wealth quintile in the HDSS or being a beneficiary of HISP for persons with severe disability. Maximum variation sampling was used to ensure representation across various age groups. Table 2 below provides the socio-demographic profile of the study participants.

**Table 2:** Participants socio-demographic profile

Participant	Age	Type of disability	Highest level of education	Marital status	Source of income	Residence	Participant description
1	70	mobility impaired	none	single	small-scale trading	urban	HDSS resident
2	32	mobility impaired	primary	single	laundering	urban	HDSS resident
3	30	mobility impaired	pre-school	married	none	urban	HDSS resident
4	35	visually impaired	secondary	separated	community health volunteer	urban	HISP beneficiary
5	60	mobility impaired	primary	divorced	small-scale trading	urban	HISP beneficiary
6	57	mobility impaired	primary	widowed	subsistence farming	rural	HDSS resident
7	48	mobility impaired	primary	married	subsistence farming	rural	HISP beneficiary
8	24	mobility impaired	none	married	small-scale trading	rural	HDSS resident
9	77	visually impaired	none	widowed	government cash transfer	rural	HISP beneficiary
10	81	visually impaired	none	widowed	government cash transfer	rural	HISP beneficiary
11	58	mobility impaired	primary	single	small-scale trading	rural	HISP beneficiary

**Fig. 1:**
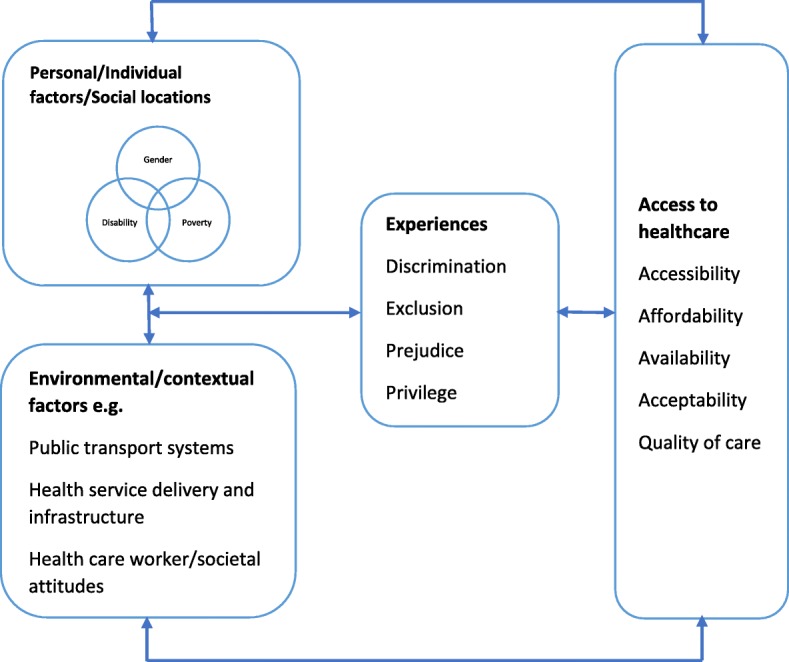
Conceptual framework

Preliminary findings from FGDs and IDIs conducted for the larger study together with the study’s the conceptual framework (Fig. 1) guided the development of semi-structured interview guides used to facilitate the in-depth interviews. Interviews were conducted at common venues within the community and at households for participants with disabilities because of their limited mobility. An informed consent form was administered to each participant and data collection began only after the participants voluntarily agreed to participate in the study. The interview guides were revised following the initial IDIs to enhance clarity and ensure a logical flow of the interview questions. All interviews were audio recorded and field notes were taken to augment the audio recordings. The IDIs lasted between 30 and 90 minutes. EK and RM^1^ collected data over a period of three months in 2017. Data collection stopped upon reaching data saturation. Table 3 outlines the distribution of interviews across the two counties.

**Table 3:** Distribution of interviews per county

Data collection method	County A		County B		Total
	HDSS	HISP	HDSS	HISP	
FGDs	2	2	2	2	8
IDIs	8	7	7	8	30

### Conceptual framework

We developed a conceptual framework (Fig. 1) adapted from the Model of Health Disparities and Disability (MHDD) [25] and intersectionality wheel diagram developed by the Canadian Research Institute for the Advancement of Women (CRIAW) [39]. The MHDD postulates that differences in health among people living with chronic diseases and various forms of impairment emerge from the interaction of personal/individual factors (biological, socio-cultural, impairment-related and psychological factors), and environmental factors (products and technologies, natural and manmade environmental changes, support systems, attitudes, health service delivery systems and services, systems and policies). These interactions influence health behaviors, quality of care and health access. The CRIAW intersectionality wheel diagram augments the MDHH by illustrating that people experience unique forms of privilege and oppression based on the complex interaction of their various social locations (personal factors in the MDHH) within a context of interconnected power structures and systems (environmental factors in the MDHH). We used the conceptual framework below to explore healthcare experiences of women with disabilities living in poverty with pro-poor health financing policies as a result of the interaction of personal factors (gender, disability, and poverty) and environmental factors, and their influence on accessing care.

#### Data analysis

Audio recordings were transcribed verbatim in MS Word and translated into English. All transcripts were verified for correctness and imported to NVivo version 10 (QSR International) for coding. Following familiarization and extensive immersion in the data, an initial coding framework was developed based on priori themes, such as privilege, discrimination, and exclusion, identified in the study’s conceptual framework. The framework was discussed extensively amongst all the authors and revised based on these consultations. Data were analyzed through a thematic approach and intersectional analysis was incorporated by identifying; individual personal factors (gender, disability, and poverty) and interactions across these factors; interactions between personal factors and environmental factors and the effects of these interactions on access to care through pro-poor health financing policies. Data were analyzed by EK with the support of all the authors.

## Results

The ability of women with disabilities living in poverty to benefit from pro-poor health financing policies was determined by the interaction of various personal and environmental factors as described below.

### Personal/individual factors

#### Women with disabilities living in poverty often opted to forgo seeking healthcare services they were entitled to under pro-poor health financing policies because of their roles in household provision and caregiving

The women with disabilities living in poverty we interviewed were responsible for financially supporting their households because most of them were single, divorced or widowed. Their roles as the sole household providers acted as a barrier to accessing care for themselves and their dependents. As the sole providers, the opportunity cost of seeking care was lost income that would negatively impact on their households. For example, some HISP beneficiaries forewent care seeking to continue earning a livelihood despite having an insurance card that facilitated access to free health services

*“I usually say, if I go to the hospital, if I leave the market, how will the children eat? Personally, how will I eat? Just that! Because I don’t have anyone else who can help me…am just the way you are seeing me now”.* Mobility impaired HISP beneficiary, County A

Women with disabilities living in poverty usually were the sole caregivers for their children and they lacked someone to assist them to watch over their children as they sought medical care.

*“Even if I decide to go to the hospital, there is no one left behind at home who knows that I have gone to the hospital so that they can help me feed the children…so I tell myself that if God knows that I am sick, I will get well.”* Mobility impaired HISP beneficiary, County A

#### Diminished mobility and the need for assistance created multiple access barriers to healthcare services offered under pro-poor health financing policies

Disabilities that imposed mobility challenges limited access to health facilities including those providing care to HISP beneficiaries. This was made worse by the long distances to some facilities contracted to provide care for HISP beneficiaries. Mobility aids such as calipers/metallic support for the legs were heavy and they made it difficult to walk the long distances to health facilities.

“*It’s far [the nearest health center] but I have to walk because sometimes I don’t have money to take a motorbike. I will just “struggle” with my leg until I reach there. Even if I get tired, I will get there and the child will get treated…I walk slowly, I can’t walk fast, I can take even one hour to get there because if I walk fast I will injure myself. This metal… this caliper is big and it’s also heavy, so I can’t walk fast, I have to walk slowly”.* Mobility impaired HDSS participant*,* County A

*“Sometimes I get very sick and sometimes I don’t have money, will I ride in this wheelchair quickly to Hospital A? This road with the trailers and vehicles and I am in my wheelchair heading to Town A, I find it difficult, it’s not easy.”* Mobility impaired HISP beneficiary, County B

To overcome mobility challenges, women with visual and mobility disabilities needed someone to accompany them to the health facility. In some cases, the accompanying person requested to be reimbursed for their assistance.

*“I go to the hospital in my wheelchair but I must get someone to help me…it takes me half an hour to get to the hospital because I ride [the wheelchair] slowly. Some people help me and others tell me “I have helped you several times, buy me “tea”. If I have KES 10, I give it to them….”* Mobility impaired HISP beneficiary, County B

Where public transport was needed, the accompanying person doubled transport costs which were difficult for women with disabilities to afford given their low socio-economic status.

*“What can prevent us from using this card [HISP]? If she gets sick and we have to go to the referral hospital, it becomes difficult to get there…Since she is blind, there must be someone to hold her from behind...from here to the referral hospital, its KES 200 for one person using a motorbike but since two people will go, I have to use KES 800 to and fro.”* Caregiver of a visually impaired HISP beneficiary, County B

Public means of transport were not disability friendly. Women with mobility and visual disabilities were either denied transport or charged a higher transport fee than abled people because they needed greater assistance when boarding alongside their assistive devices and this process was viewed to be time-consuming.

“*It’s difficult to use public means of transport because they don’t like putting the wheelchair in the vehicle…. it wastes their time carrying it from the ground and putting it on top of the vehicle and then they will have to remove it, it’s difficult, sometimes they are in a hurry to go and transport people. They only agree if we are going long distances…if we are going to a place that costs KES 100 and above or KES 200 that’s when they allow us to board but if it’s a short distance they can’t agree”.* Mobility impaired HISP beneficiary, County B

*“The vehicles don’t stop; they refuse completely because assisting us to board is a challenge “*Visually impaired HISP beneficiary, County A

*“From here to hospital A they [abled people] pay KES 50 but I am transported [using a motorbike] with KES 100 to get to hospital A, to and fro KES 200…It makes me wonder if I am not a human being or what could be wrong with me? Even if you are walking along the road…when the motorbike riders find you they say “look at this “problem” in front of me, it wants to cause me trouble” while you were just standing by the roadside”.* Mobility impaired HISP beneficiary, County B

Without someone to accompany them to a health facility and without money to cater for transport costs to the health facility, some women with disabilities living in poverty could not access care. For example, some of the women we interviewed reported failing to access antenatal care and delivering at home despite the existence of the free maternity program.

“*I was alone in the house and the hospital was far. The person who would have taken me to the hospital was not near but God helped me and I delivered without any problem. They found me when I had finished delivering”.* Mobility impaired HDSS participant, County A

*“This time around she went [to the ANC clinic]. The other times she didn’t go because there was no one to push her [wheelchair]. Right now she goes because her child pushes her [wheelchair]”.* Mobility impaired HDSS participant, County B

### Environmental factors

#### Disability unfriendly health facilities limited the extent to which women with disabilities living in poverty could benefit from healthcare services offered through pro-poor health financing policies

Health facilities offering care under pro-poor health financing policies were not structured to meet the needs of women with disabilities. First, absence or shortage of sign language interpreters and guides in public health facilities led to delayed or lack of care for women with disabilities living in poverty.

*“Those who don’t talk, those with hearing impairment, you will find that the sign language interpreter maybe is not in the center or he/she is there but they are alone and maybe they are held up somewhere else. So there are those who have been complaining that they go to the hospital and they are not treated because maybe the sign language interpreter was not there that day”.* Visually impaired HISP beneficiary, County A

*“Sometimes you will get stranded even before you get to the doctor, you don’t know where you are and you don’t know where to start because you don’t have someone to guide you. So there should be someone to guide you so that you can get to the doctor.”* Visually impaired HISP beneficiary, County A

Second, health facilities’ layout limited mobility of women with visual and mobility disabilities due to the absence of ramps.

“*There are some places I use the wheelchair comfortably but there are other places that have stairs so I can’t use the wheelchair…the paths should be straight…. without stairs where one can fall over”.* Mobility impaired HISP beneficiary, County B

“*There are no ramps…. I have to use the stairs and I am totally blind; I can’t move without someone to assist me”.* Visually impaired HISP beneficiary, County A

Third, health facilities lacked disability friendly facilities and equipment. Toilet doors were too narrow to allow for wheelchair access and where toilets were accessible, the toilet seats were low and this made it difficult for women with disabilities to utilize them. Lack of adjustable beds necessitated more assistance from healthcare workers to enable women with disabilities to utilize hospital equipment.

*“If I know am going to the hospital, I don’t drink anything that can make me want to go to the toilet because if I go to the toilet, I will have to leave my wheelchair at the door…the toilet doors are narrow…I don’t use the toilet until I get back home…the small wheelchairs they use in the hospital for patients can fit but I use a tricycle it’s a bit wide it can’t fit through the toilet door….we need toilets with a wide door, also when you enter the toilet seats shouldn’t be low, they should have high toilet seats so that if you enter the toilet you just sit on it and when you are through you go back to the wheelchair.”* Mobility impaired HISP beneficiary, County B

*“For sure those beds are not disability friendly…I suffered…I asked them if there was a way it could be pressed to come down so that I can climb onto the bed and then we lift it up again but it was difficult so I asked them about my friends with mobility disabilities, what do those on wheelchairs do... they said they have to carry them and lift them onto the bed”.* Visually impaired HISP beneficiary, County A

#### Prejudice and negative attitudes by healthcare workers and other health system workers disempowered women with disabilities living in poverty and discouraged them from accessing the care they were entitled to under pro-poor health financing policies

Women with disabilities felt that healthcare workers had negative attitudes towards them because of their disability. One of the reasons was the additional assistance that they required because of their disability. Some healthcare workers were unwilling to offer the extra assistance needed by women with disabilities.

“*You can find a nurse asking you when you are having labor pains ‘How will I attend to you? where will I start, where will I finish?’ For example, I have a visual disability and also I have to be lifted to the bed first or be shown where the bed is and so forth….so you find, it’s like they don’t understand”.* Visually impaired HISP beneficiary, County A

*“Even if you tell the doctors to assist you with carrying water, they ask you “why don’t you tell your husband to carry for you, you are disturbing us, just stay there you will help yourself. I felt like they were looking down on me… I couldn’t be able to even open that pipe [urinary catheter] to drain the urine, discarding it was also a problem”.* Mobility impaired HDSS participant, County B

Some healthcare workers also questioned their right to be sexually active and their right to have children.

“*They [healthcare workers] told her it’s not good for her to give birth and it’s not good for her to have sex. Because she has a problem with her legs it’s not good to have sex with men, it’s not good for her to give birth.”* Mobility impaired HDSS participant, County B

*“They feel we don’t have a right to get children. I also have a friend [with a disability] who went to the antenatal clinic and she was asked “even you?*” Visually impaired HISP beneficiary, County A

These staff attitudes resulted in a bad patient experience that discouraged the women from seeking healthcare services offered under pro-poor health financing policies.

*“When I went to deliver, the healthcare workers don’t view you as a person who needs their help. No! you face difficulties all by yourself, it’s even hard for them to attend to you…. I told myself if I continued to give birth, I would die.”* Mobility impaired HISP beneficiary, County A

*“I tell myself, disabled people don’t have someone who will serve them quickly. Even if I go to the hospital, they won’t attend to me, I just stay at home”* Mobility impaired HISP beneficiary, County A

Despite feeling dissatisfied with the quality of care provided, women with disabilities felt disempowered to speak out or raise complaints about the care they received. This was because they felt that health workers did not take their complaints seriously.

*“I feel that the healthcare worker is mistreating me because I am disabled, so overall, we [women with disabilities] don’t like speaking up”.* Mobility impaired HDSS participant, County B

Negative stereotypes against women with disabilities in society led to their exclusion from public participation forums. They reported that they were not invited to take part in public forums because they were perceived to be uneducated and could therefore not participate effectively. Some women with disabilities were also not informed of such meetings because people felt that they could not get to the meeting venue quickly because of their limited mobility and they would, therefore, require their assistance to get there. This limited their awareness of health services and their opportunity to contribute to public participation forums related to health service provision.

*“They have never taken me for the awareness rising forums [for HISP]. They say they want someone who is educated and they leave me behind… They say we are selecting people who talk and those who answer questions the right way… I wonder, “they should invite me one day for the meeting and then they will find out if I will not answer [the questions]”.* Mobility impaired HISP beneficiary, County A

*“Sometimes the chief calls for a meeting but no one will inform you, you will just hear that people went for a meeting and they came back. No one informs you because you don’t have the ability to get there quickly, you will disturb people”.* Mobility impaired HISP beneficiary, County B

*“We are not invited, for example, if there is a public meeting, there are meetings of certain kinds that we are excluded a lot from . That’s why you find that if we go to hospitals we don’t know even where to start…we have never been invited and told this and that or gathered together with others and told these are the developments, so you find some of us are discriminated against”.* Visually impaired HISP beneficiary, County A

However, some respondents reported that at times, clients with disabilities received preferential treatment from healthcare workers in public, rather than private, health facilities because of their disability. For instance, some healthcare workers in public healthcare facilities at times allowed people with disabilities to jump the long queues and hence be attended to before other clients..

“*Even if I get there [health center] at whatever time…they will have mercy on me and treat my child…there is a day my child was sick and I got there when they were closing. So they looked at how I was and they said “let’s help her so that she doesn’t go back with the sick child. She walks slowly and she can’t get here quickly.” So those who were there remained behind and they helped me. The government health center helps even a disabled person like me…They will help me because they know am not able. I can’t go to these private ones because they won’t help me, they want money*”. Mobility impaired HDSS participant, County A

Also, most of the women with disabilities who were HISP beneficiaries stated that they received their insurance cards on the same day upon registration compared to persons without a disability who had to wait for weeks or months to obtain the insurance/HISP card.

*“They [NHIF officials] are in a multi-story building, but I can’t climb the stairs, so I just stayed outside, they were serving me from outside. They told me it’s not good for me to go back, I got that HISP card the same day”.* Mobility impaired HISP beneficiary, County B

## Discussion

Our findings reveal that despite the existence of health financing interventions that target poor individuals in Kenya, such as the free maternity policy, the health insurance subsidy programme for the poor, and user fee removal policies, women with disabilities living in poverty faced unique access barriers as a result of the interaction between personal and environmental factors. Being the primary household providers and caregivers, women with disabilities living in poverty reported they often forwent care to sustain their families’ livelihoods reflecting how gender roles and poverty intersected to limit their access to care. It has been documented elsewhere that many women can lack time to seek medical care and to go for regular check-ups because of their time-consuming childcare and household responsibilities in addition to engaging in formal economic activities to sustain their households [40]. Women are therefore more likely to report poorer health than men [17]. This is exacerbated for women with disabilities, who in addition to ‘routine’ gendered childcare and domestic responsibilities, they face further unique barriers when accessing care as illustrated in this study.

Disability and poverty interacted to influence access to care provided under pro-poor health financing policies. Due to limited mobility, women with visual and mobility disabilities needed someone to accompany them to health facilities. The accompanying person led to greater transport costs which were difficult for the women to afford because of their meager earnings from informal employment. These factors in addition to disability unfriendly public means of transport contributed to women with disabilities foregoing care. These findings are consistent with literature from low and middle-income countries which shows that transport costs for persons with disabilities can be highly prohibitive [22, 41–44]. The latter is compounded by many requiring someone to accompany them to health facilities [17], and having low or no source of regular income [45]. In addition, evidence from rural Northern Namibia shows that public transport providers also found it cumbersome to transport people with disabilities especially those with bulky assistive aids such as wheelchairs [42]. In this study, limited mobility and unaffordability of transport costs to health facilities illustrate the complex bidirectional relationship between poverty and disability [17, 46] whereby disability increases the risk of becoming poor and vice versa [15, 18]. People with disabilities have a lower likelihood of being employed [17, 20, 41] and if employed they tend to earn less than abled persons [17]. According to the World Health Survey, the employment rate for men with disabilities was more than twice that of women with disabilities in 51 countries [18]. Disability also limits one’s income-earning ability [15], it increases health care expenditure [15, 18, 47], and increases the risk of impoverishment [15]. Furthermore, there is evidence that unavailability of someone to accompany disabled people [44, 45] or lack of means of transport [41] to health facilities can lead to delayed [45] or failure to seek care [41, 44]. Our findings support literature that shows that despite people with disabilities having increased need for healthcare services [17, 21–23], those needs are more likely to be unmet [23–25] compared to people without disabilities.

Intersections of disability and disability unfriendly health system structures created multiple access barriers. Healthcare facility layouts, equipment, services and human resources were not structured to adequately meet the healthcare needs of women with disabilities and this limited the extent to which women with disabilities living in poverty benefited from pro-poor health financing policies. This is in keeping with other studies which reported the absence of sign language interpreters [24, 41, 44] and health care providers’ impatience with people with speech difficulties [24, 44]. Communication barriers are reported to have led to mistranslation of patient symptoms [41], and delayed [24] or forgone care [24, 43, 48]. Studies have also documented the unfriendly nature of health infrastructure - also described as ‘environmental discrimination’ [17] – as evidenced by lack of ramps [43], narrow doors which prevented wheelchair access [41, 43], disability unfriendly delivery beds [43], examination tables [24, 48] and equipment [48], and inaccessible [45] or lack of washrooms for people with disabilities[41, 43].

Women with disabilities face multiple layers of discrimination because of interactions between gender and disability [47, 49, 50], leading to poorer access to healthcare services compared to women without disabilities [21, 51]. Our study findings revealed that negative healthcare worker attitudes and prejudice against women with disabilities resulted in adverse patient experiences which discouraged them from seeking care. Our findings are similar to studies where women with disabilities reported that healthcare providers were highly insensitive [43, 52], rude [24, 41, 43, 44], seemed to lack awareness about their needs [24, 43, 45], and were surprised that women with disabilities were sexually active [45, 52]. People with disabilities also reported being denied treatment [22, 44, 53]. However, one study done in rural Namibia showed that people with disabilities were impressed by health care workers skills and training [41]. In this study, the effects of negative health care worker attitudes were compounded by the fact that women with disabilities felt disempowered to complain about the poor quality of care provided. According to the WHO global disability action plan, 2014–2021, people with disabilities face unique barriers when it comes to expressing themselves and accessing information [20]. However, some women with disabilities got some privileges, such as being assisted to skip hospital queues and being issued their insurance card immediately upon registration to HISP. This was in line with the Kenya Persons with Disability Act, 2003 which recommends that persons with disabilities should receive prompt health service delivery [27]. Similarly, some facilities in Malawi allowed people with disabilities to receive care without having to queue [44].

One of the emerging issues from the study was that disability and education intersected to influence healthcare experiences. Negative stereotypes against women with disabilities in society led to their exclusion from public participation forums and this limited their awareness of health services. One of the factors contributing to the exclusion was the perceived low education status of women with disabilities. Majority of the women who took part in the study had not received any form of education or they had attained primary level education. This is consistent with literature which shows that women and girls with disabilities tend to be less educated compared to men with disabilities or women without disabilities [54]. A relationship exists between disability, poverty and low level of education [15, 17]. Lack of education is recognized as one of the factors contributing to poverty among people with disabilities and households living in poverty may invest less in educating children with disabilities [17]. Our study findings are also supported by literature which shows that interactions between poverty, gender, and disability [47] and broader contextual factors [17] limits the opportunities for disabled people, especially women, to participate fully both economically and socially within the society [17, 47]. In most societies, people with disabilities living in poverty are the least vocal and most vulnerable populations and this limits their access to resources which in turn limits their agency and ability to fight for their rights [47]. For women with disabilities, this is compounded by gender inequities which further limit women’s agency and access to financial and social resources.

The study findings highlight the importance of a holistic systems approach to improving access to healthcare services. Implementing health financing reforms alone, without attendant non-finance reforms, is not sufficient to achieve increased access. This is even more important for reforms that target the poor since they often have other vulnerabilities in addition to poverty. Our study findings also expound on the importance of applying an intersectional approach which posits that social factors intersect in complex ways to sometimes simultaneously create experiences of privilege and disadvantage for an individual. In the Kenyan case, gender (being a woman) and disability add additional layers of vulnerability that need to be taken into account in designing pro-poor health financing interventions. Access to healthcare for people with disabilities is a reflection of equitable and gender-sensitive health systems [49]; and without equitable and gender-sensitive health systems which address these access barriers, universal health coverage will not be achieved.

We make several recommendations. To address geographical access barriers, for women with disabilities living in poverty, resulting from limited mobility and inability to afford high transport costs, the NHIF should ensure that healthcare facilities that are contracted to provide healthcare services for beneficiaries of pro-poor interventions, such as HISP and the free maternity programme, are near the locations where they reside. Also, the NHIF and county governments should consider incorporating transport vouchers in pro-poor health financing mechanisms to alleviate the increased transport costs that persons with disabilities face. The National and County governments should work together with the NCPWD to enforce laws that ensure that public means of transport are disability friendly as stipulated in the Persons with Disabilities Act, 2003.

To improve health system responsiveness to the needs of people with disabilities, county governments need to: build cultural competence in health service delivery, especially for maternal and reproductive health services, to ensure it promotes dignity and is non-discriminatory; ensure availability of sign language interpreters, guides and hospital assistants and ensure that hospital layouts, equipment, and facilities are disability friendly. County governments and health facility managers should also strengthen accountability mechanisms such as client feedback and grievance redress mechanisms to ensure that people with disabilities living in poverty and other marginalized groups are empowered to engage in public participation. An inter-sectoral response is needed to sensitize communities on the needs of people with disabilities living in poverty in order to reduce stigma and discrimination and strengthen family and community support structures. Until such interventions are implemented, pro-poor health financing reforms will continue to exclude some of the most marginalized including women with disabilities living in poverty.

### Study limitations

The use of in-depth interviews facilitated an in-depth exploration of the study topic. However, focus group discussions might have been useful to obtain multiple views on respondent’s experiences, attitudes, and beliefs within a group context. The research team was also unable to employ a sign language interpreter due to cost constraints and therefore we managed to interview women with mobility and visual disabilities only. Future studies should consider including women living in poverty with all forms of disability in order to increase the knowledge base on the different barriers they face when accessing care based on the nature of their disability.

## Conclusions

Women and poor people are disproportionately affected by disability [20] and when gender (being a woman ), disability and poverty intersect, it results in multiple layers of discrimination [47]. In Kenya, women with disabilities living in poverty experienced advantages and disadvantages when seeking care under pro-poor financing reforms that targeted them. This was as a result of interactions of personal factors such as gender, disability, and poverty, with environmental factors such as disability unfriendly transportation systems and health systems structures and negative healthcare worker attitudes. In an effort to achieve equitable health care for all, health systems need to address the unique barriers that people with disabilities face when accessing healthcare. This should entail incorporating an intersectional and gender lens to enhance understanding of the varying degrees of vulnerabilities in accessing health care across social groups, as a result of the interaction of their social locations, such as gender, poverty, and disability, with the underlying socio-economic and political structures.

## Abbreviations

CRIAW: Canadian Research Institute for the Advancement of Women
FGD: Focus Group Discussion
HDSS: Health and Demographic Surveillance System
HISP: Health Insurance Subsidy Programme
IDI: In-Depth Interview
MHDD: Model of Health Disparities and Disability
NCPWD: National Council for Persons with Disabilities
NHIF: National Hospital Insurance Fund
SDH: Social Determinants of Health
UHC: Universal Health Coverage
WHO: World Health Organization
